# A Batch of Interesting Questions

**Published:** 1857-08

**Authors:** 


### A BATCH OF INTERESTING QUESTIONS.

We find in the New Orleans Medical News and Hospital Gazette—

"An Interesting Question :"—Is it a fact that the Massachusetts Medical College will graduate "persons whom they know will practice Homeopathy ?" And by the by, does the Massachusetts Medical Society ever expel members who edit quack Journals ?" To which we would add—Is it true that there is a medical college in the United States calling itself respectable, that has been guilty of sending out its tickets (or an equivalant,) to be sold in the market for what they will bring ?

